# Advanced practice provider–led clinic for care transitions in newly diagnosed venous thromboembolism: establishment and utilization

**DOI:** 10.1016/j.rpth.2023.100198

**Published:** 2023-06-01

**Authors:** Cassiopeia Frank, Raj Kasthuri, Nigel S. Key, Micah Mooberry, Samuel R. Wilson, Stephan Moll

**Affiliations:** University of North Carolina School of Medicine, Chapel Hill, North Carolina, USA

**Keywords:** advanced practice provider, anticoagulants, care transition, thrombosis, venous thromboembolism

## Abstract

**Background:**

Patients with suspected or newly diagnosed venous thromboembolism (VTE) are often referred to the emergency department (ED) for management, where anticoagulation is initiated. However, when the patient is judged to be suitable for outpatient management, counseling and follow-up specialty care are frequently suboptimal.

**Objectives:**

To establish an advanced practice provider (APP)–led rapid follow-up clinic to improve transitions of care for patients with newly diagnosed deep vein thrombosis or low-risk pulmonary embolism and to provide continued specialty care and support, including management of complications and medication access issues.

**Methods:**

In order to address this gap in transition of care, we developed an APP-led clinic with a mandate to improve quality and safety in the outpatient setting for patients with acute VTE.

**Results:**

In the first 2 years, a total of 234 patients were evaluated, of whom data were standardized and reviewed for 229. Utilization steadily increased over time, with at least 10% of patients requiring financial medication assistance over both years. Seventy-two percent of patients were referred from the ED in the first year and 59% in the second year, and referrals from non-ED outpatient specialties increased. Data on deviations from standard care identified in referred patients were collected in the second year and found in 19 (12.7%) of cases. These included unnecessarily prescribed or changed anticoagulants, dosing errors, misclassification of thrombosis, and other deviations. Patient demographic data also demonstrated increasing diversity of the patient population over time, with increased utilization by Hispanic and African American patients in the second year. This highlighted the need for better patient education material translations into Spanish, which is a future aim.

**Conclusion:**

In summary, the APP-led VTE Transition Clinic was feasible and grew quickly in utilization, diversity of referrals, and diversity of patients served.

## Essentials

• There is a need for transitions of care in patients with newly diagnosed venous thromboembolism.
• An advanced practice provider–led clinic was designed to address this need at University of North Carolina Health.
• Utilization of the care transitions clinic grew over time, serving >200 patients in 2 years.
• The established model was feasible and evolved to serve increasingly diverse referral sources.

## 1 Introduction

Patients with suspected or newly diagnosed venous thromboembolism (VTE) are often sent to the emergency department (ED) for management, where they are started on anticoagulation. Time is limited for counseling and continued support for patients on new prescribed anticoagulation, as is access to specialty care.

Evidence supports treating most patients with VTE as outpatients [1]. Resources exist to support safe and comprehensive transitions of care in these patients, such as those provided by the Anticoagulation Forum [2]. However, there is hesitancy among some healthcare providers to manage patients with these morbid acute conditions on high-risk medications without appropriate follow-up. In the era of direct oral anticoagulants, the financial barriers associated with appropriate anticoagulation further complicate management. Rapid access to specialized care is limited, and many patients do not have access to specialized follow-up immediately after discharge.

Transitions of care provided by hematology providers allow for immediate assessment of appropriateness of anticoagulation, counseling on anticoagulation risks and benefits, and assistance in navigating barriers to medication access. Additionally, connecting to a specialty provider provides an ongoing resource to prevent repeat ED visits for complications that can be managed as an outpatient, such as continued pain or nonmajor bleeding. An initial discussion regarding the etiology of the episode can help patients develop a framework for their new diagnosis and long-term implications. Specialty providers are also able to help with procedural planning and care for patients in special populations, such as pregnant women, patients on hormone therapy, and patients with cancer.

A similar transition of care gap was identified in stroke follow-up and prevention of recurrent stroke at a midwestern Comprehensive Stroke Center [3]. In 2014, in response to this gap, an advanced practice provider (APP)–led clinic was developed, wherein patients were directly scheduled with an APP at discharge for stroke [3]. This clinic demonstrated the feasibility of an APP-led model of care transition for a high-risk condition with the potential for significant utilization and growth [3]. A similar APP-led transitions model in atrial fibrillation demonstrated not only feasibility but also improved adherence to American College of Cardiology/American Heart Association clinical performance and quality measures when patients were referred for specialty care compared with standard follow-up [4]. These patients were more likely to have screening for other relevant conditions and be prescribed anticoagulation appropriately [4].

Transitions of care in VTE by nonphysician providers have also been previously demonstrated by DiRenzo et al [5]. In this model, patients discharged from the ED were managed by an outpatient primary care physician or a pharmacist [5]. In this study, patients managed by pharmacists had similar safety outcomes when compared with primary care physician–managed follow-up [5].

## 2 Methods

An APP-led acute VTE clinic, “VTE Transition Clinic,” located within the Benign Hematology specialty clinic at the University of North Carolina (UNC) was established in January 2020 to provide transitions of care from outpatient or ED providers to hematology. Formal review of data did not begin until August 2020. The initial structure included 1 appointment reserved each weekday morning. For purposes of scheduling, this clinic has its own scheduling template without an assigned provider, and patients are later moved to the schedule of the treating provider. This allows free scheduling by referring locations without concerns about interference with other visits on an existing provider schedule.

The clinic was advertised to referring providers primarily through face-to-face interactions with the APP coordinator, who presented the clinical model as a guest at faculty and staff meetings throughout the UNC Health system. These meetings involved education on appropriate referrals and how to reach the clinic. Patients with newly diagnosed VTE can be referred by any UNC-affiliated provider. An algorithm was made available to the ED providers for determining appropriateness for outpatient management and referral. The eligibility criteria were modified from the Hestia criteria and applied to both deep vein thrombosis (DVT) and low-risk pulmonary embolism (PE) [6]. A triage system with direct pager and phone line (“1-DVT”) is available during business hours. For patients referred after-hours through the ED, direct scheduling is available 24 hours a day through a patient access phone line more broadly utilized by UNC Health.

The hematology group includes 2 APPs who see classical (nonmalignant) hematology patients (including anemia and other disease groups outside of thrombosis) but reserve 1 slot each per day, in addition to their standard clinical load, for VTE transition patients. Patients are seen by the APP (physician assistant or nurse practitioner) with on- or off-site hematologist oversight. The APPs within this clinic practice within their scope of practice as outlined in their collaborative practice agreement, per requirements of the state of North Carolina. Pharmacist support is available for questions about drug interactions, medication access, and other needs through on- or off-site communication with a clinical practice pharmacist who practices with similar oversight. Patients are seen only by the APP, but collaborating physicians are available for issues as they arise.

Patient education and counseling on diagnosis and anticoagulation, including duration, is provided to patients during their visit. Patients are evaluated for risk factors that may have contributed to the VTE, including a detailed personal and family medical history. A review of risk factors for VTE and patient education materials regarding risk factors and signs and symptoms of VTE are provided. Patients and their families and caregivers are counseled on bleeding risks associated with anticoagulation and signs and symptoms of bleeding. Additionally, bleeding risk is assessed and any potential major risk factors are identified (concomitant antiplatelet use, fall risk, and impaired renal function) for further management and risk mitigation. Laboratory testing of a complete blood count and creatinine is often included in the risk assessment, and other testing is pursued on a case-by-case basis (liver function testing, repeat imaging, and thrombophilia testing). Patients are generally scheduled for follow-up for longer-term anticoagulation planning at 3 to 6 months after the initial visit. Patients are provided printed VTE education materials, including the Clot Connect brochure, formerly available on www.clotconnect.org [7]. Following their initial appointment, patients have access to nurse and provider assistance for bleeding complications, anticoagulation management for surgical/procedural planning, medication access, or other complications.

Data were collected through chart review and recorded by the APP providing care. Details regarding demographic information, diagnosis, referring information, and deviations from the standard of care were documented by the APP who provided care. In the first year, the focus was on clinic establishment and streamlining referral triage and access to the clinic. In the second year, deviations from protocol were documented in real time for all cases where the APP determined a deviation from standard of care had occurred requiring a change in plan of care. These were divided into 4 categories. The first category included patients in whom anticoagulation was started unnecessarily (ie, the patient did not truly have an indication for anticoagulation) or a change in anticoagulation was made unnecessarily (ie, the patient was thought to have had a recurrent VTE despite anticoagulation but had not). The second category included dosing errors of any kind (incorrect frequency, dosage, or duration of given dose/frequency). The third category was misclassification, which refers to cases where a thrombus was classified incorrectly as deep, superficial, proximal, or distal. The last category included other situations, including those where inpatient treatment was recommended in a patient who had been deemed suitable for outpatient management.

Data collection and interpretation utilized standard descriptive analytics, including median, median, and range values. Demographic changes from year 1 to year 2 were analyzed using a Fisher’s exact test.

## 3 Results and Discussion

In the 2-year period since inception, a total of 234 patients were seen in the clinic. Of these, 84 were seen in the first year (August 24, 2020, to July 31, 2021) and 150 were seen in the second year (August 1, 2021, to July 31, 2022). Data were available and reviewed for 229 of the 234 patients. Data analysis is based on these 229 patients.

Patients from throughout the statewide UNC Health system were seen in the clinic, with the majority referred from UNC Medical Center in Chapel Hill, North Carolina, which includes a >950 bed hospital. The exact number of DVT and PE diagnoses made in the healthcare system is unknown, but in the second year period (August 1, 2021, to July 31, 2022), 304 patients were diagnosed with DVT alone by the main hospital peripheral vascular laboratory. A total of 113 DVT-only patients were seen in the clinic during that time period.

The median age remained similar, 55 years (SD, 17.47 years) in year 1 and 55.5 years (SD, 18.44 years) in year 2 (Table). African American patients made up 20.3% of the patients in year 1 and 25.3% of the patients in year 2 (Table). Hispanic patients represented 3.8% of the patients in year 1 and 9.3% of the patients in year 2, which was not a statistically significant change (Table).

**Table:** Patient demographics of the DVT Transition Clinic Program from year 1 (2020 to 2021) and year 2 (2021 to 2022).

Patient demographics	2020-2021	2021-2022
Age (y)		
Range	22-88	18-92
Average	55.3	54.9
Median	55	55.5
Sex, n (%)		
Male	41 (51.9)	84 (56)
Female	38 (48.1)	66 (44)
Race, n (%)		
African American	16 (20.3)	38 (25.3)
Asian	1 (1.3)	3 (2)
Caucasian	55 (69.6)	92 (61.3)
Hispanic	3 (3.8)	14 (9.3)
Unknown or other	4 (5.1)	3 (2)

DVT, deep vein thrombosis.

**Figure 1:**
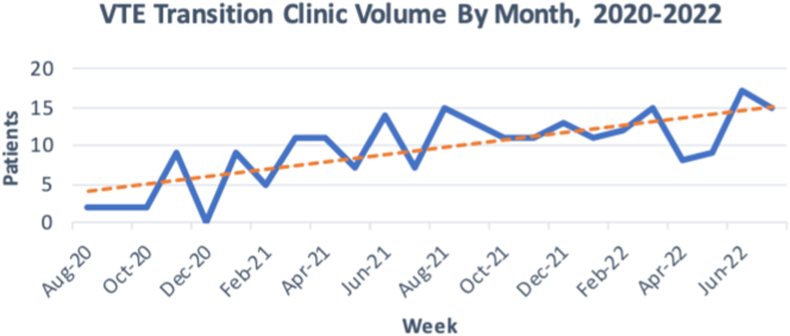
Patient volume by month from August 2020 through August 2022. VTE, venous thromboembolism.

**Figure 2:**
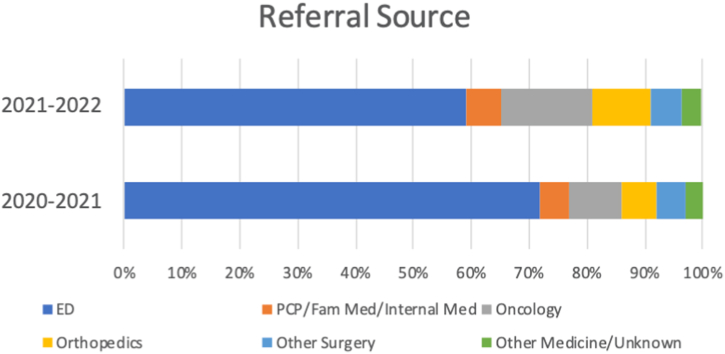
Referral source by specialty or department in 2020 to 2021 compared with 2021 to 2022. ED, emergency department; Fam, family; Med, medicine; PCP, primary care physician.

Utilization steadily increased over the 2 years (Figure 1). In the first year, 72% of patients were referred from the ED (Figure 2) and 12.7% of all patients required financial assistance of some kind. In the second year, 59.3% of the referrals were from the ED (Figure 2) and 10% of all patients required medication assistance. Medication assistance included providing a copay card, assisting with enrollment in pharmaceutical manufacturer assistance programs, enrollment in UNC-specific pharmacy assistance programs, or other assistance depending on patient need and circumstance.

Data on deviations in protocol were not collected in the first year, but in the second year, significant deviations from protocol were identified in 19 (12.7%) cases. These deviations included 8 circumstances in which anticoagulation was found to be unnecessary and was discontinued or a change had been made unnecessarily to previous anticoagulant therapy and the previous agent was resumed. In 4 cases, dosing errors were identified, and in 4 additional cases, the diagnosis was found to be misclassified. The remaining 3 were other deviations. The median time to appointment in the first year was 4 days, and in the second year, it was 5 days (including weekend days). The APP has access to hematology physician providers in the clinic or by phone for consultation at all times. In the first year, an MD provider saw the patient in a shared visit, where the MD independently assessed the patient, only once. Due to the rare need for this, shared visit data were not collected in the second year.

The VTE Transition Clinic grew quickly in its first year and continued to grow in the second year, nearly doubling in utilization. In the first year, the median time to visit was 4 days, and the program’s aim was to decrease this. However, with increased utilization, this metric increased to 5 days. As a consequence, a second APP was hired to improve access. A second visit is now available daily in the afternoons to allow increased capacity and more rapid access to the clinic. Additionally, 2 medical assistants within the clinic have been trained to help provide previsit patient outreach and ensure communication throughout the transition of care.

The total number of referrals increased from year 1 to year 2, including ED referrals (56 in year 1 and 89 in year 2). However, the types of providers referring to the clinic diversified in the second year, with more referrals from surgical and medicine specialties and a lower overall percentage of ED referrals. This finding supports progress toward our ultimate goal to encourage outpatient providers who diagnose a DVT or low-risk PE suitable for outpatient management to bypass the ED and refer directly to the VTE Transition Clinic. Expanding the network of outpatient providers using the clinic is a future goal, and outreach and referral pathways for care transitions from outpatient providers are currently being pursued. This may be achieved through continued outreach and education, establishment of direct rapid referrals from outside systems, as well as incorporation of telemedicine to reach rural and underserved areas. The data show that deviations from standard of care, including unnecessary utilization of anticoagulants, were identified and risks were potentially mitigated through the use of this clinic. This finding further supports an ongoing need for follow-up specialty care and expanded access for referring providers.

The patient demographic data show an increase in use by African American and Hispanic patients. The increase in use by Hispanic patients has highlighted the need for better patient education materials translated to Spanish. This prompted efforts to both update existing patient education materials and have these materials translated and available in Spanish.

**Figure 3:**
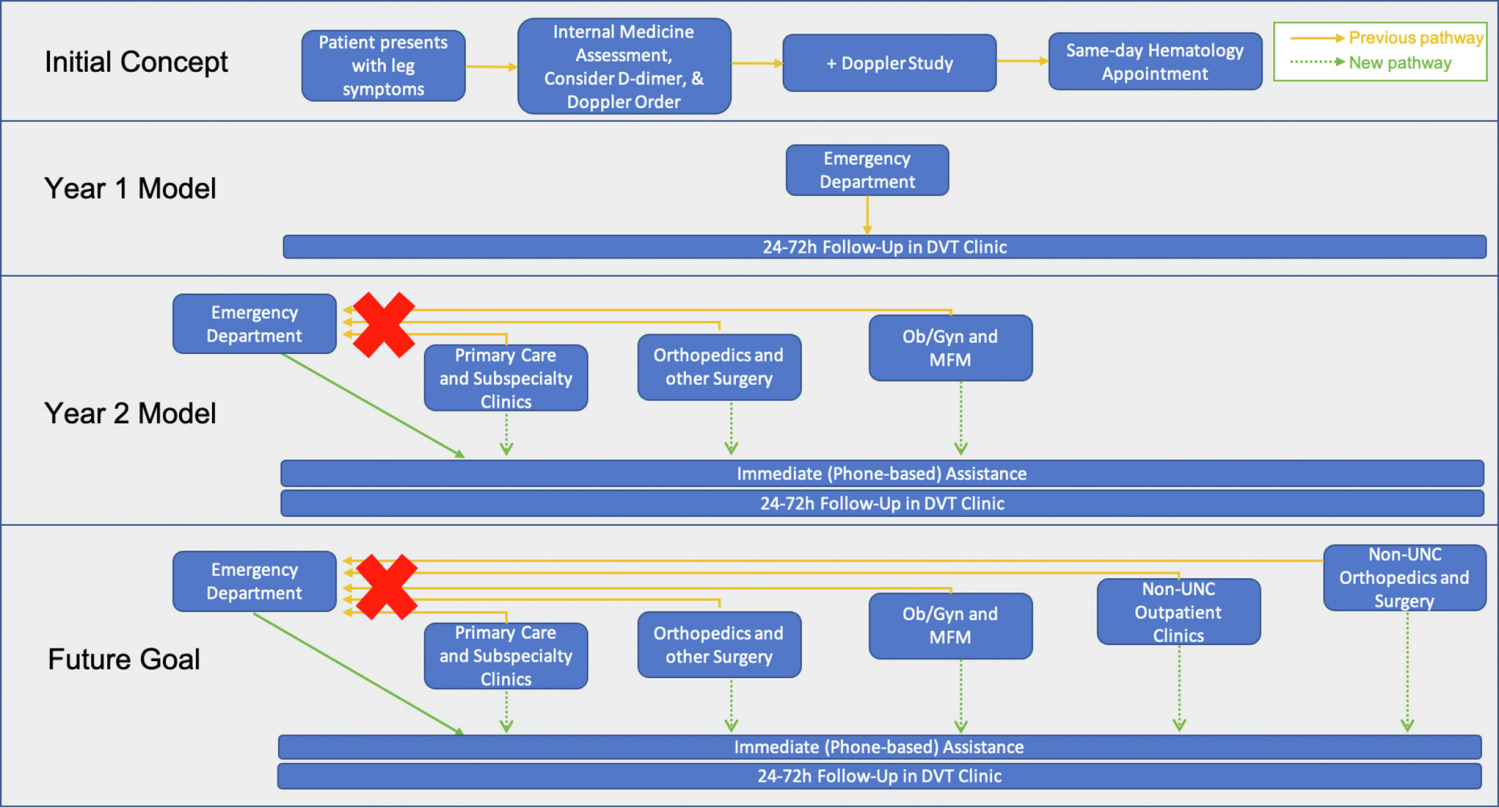
Flowchart describing the change in care model from initial concept to current model and future goals. DVT, deep vein thrombosis; Ob/Gyn, obstetrics/gynecology; MFM, maternal fetal medicine; UNC, University of North Carolina.

It is notable that the described thrombosis transitions clinic model was developed after several prior iterations involving on-site diagnosis of DVT in a “painful leg clinic” were unsuccessful (Figure 3). Collaborations with internal medicine and vascular surgery required in initial attempts, which would have started at the point of symptoms rather than confirmed diagnosis, proved logistically too difficult. The current clinic focuses on the hematology-led aspects of care rather than a comprehensive model starting with diagnosis. Additional barriers to continued success include the required education of new providers and trainees, changing referral pathways and dynamics in the healthcare system, and turnover of staff trained to schedule into the clinic.

In summary, since its inception, the APP-led VTE Transition Clinic has grown in utilization, diversity of referrals, and diversity of patients served, with new focus on patient education tools and expanding access.
